# Genome Context Viewer (GCV) version 2: enhanced visual exploration of multiple annotated genomes

**DOI:** 10.1093/nar/gkad391

**Published:** 2023-05-19

**Authors:** Alan M Cleary, Andrew D Farmer

**Affiliations:** National Center for Genome Resources, 2935 Rodeo Park Dr E, Santa Fe, NM 87505, USA; National Center for Genome Resources, 2935 Rodeo Park Dr E, Santa Fe, NM 87505, USA

## Abstract

The Genome Context Viewer is a web application for identifying, aligning, and visualizing genomic regions based on their micro and macrosyntenic structures. By using functional elements such as gene annotations as the unit of search and comparison, the Genome Context Viewer can compute and display relationships between regions across many assemblies from federated data sources in real-time, enabling users to rapidly explore multiple annotated genomes and identify divergence and structural events that can help provide insight into evolutionary mechanisms associated with functional consequences. In this work, we introduce version 2 of the Genome Context Viewer and highlight new features that enhance usability, performance, and ease of deployment.

## Graphical Abstract

**Graphical Abstract:**
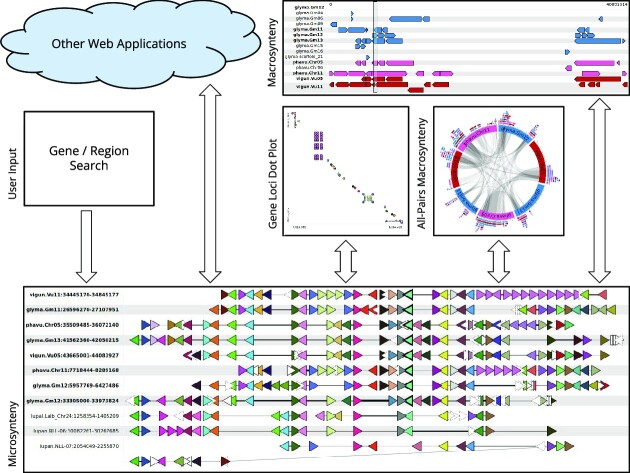

## INTRODUCTION

Multiple annotated whole genome assemblies within a clade or species are now widely available due to improvements in sequencing technologies, assembly and annotation algorithms, and computational infrastructure (1,2). Researchers that work with such collections that span large phylogenetic distances or exhibit structural variation are often concerned with functional content and the genomic contexts in which it occurs, rather than all sequence base-pair level variation. Identifying such content can be computationally challenging and is made more difficult when the genomes of a collection are provided by disparate databases.

In this work, we present version 2 of the Genome Context Viewer (GCV) – an open-source web application that uses the functional annotations of genes to perform on-demand federated synteny analysis of collections of genomes (3). By using functional annotations as the unit of search and comparison, GCV can compute and display multiple regions across several assemblies from different databases in real-time. This enables users to rapidly explore multiple annotated genomes and identify divergence and structural events of interest. Using GCV’s support for interoperability with other web applications, genes, regions, and annotations of interest can be further interrogated in other, database-specific tools.

## GCV OVERVIEW

**Figure 1.**
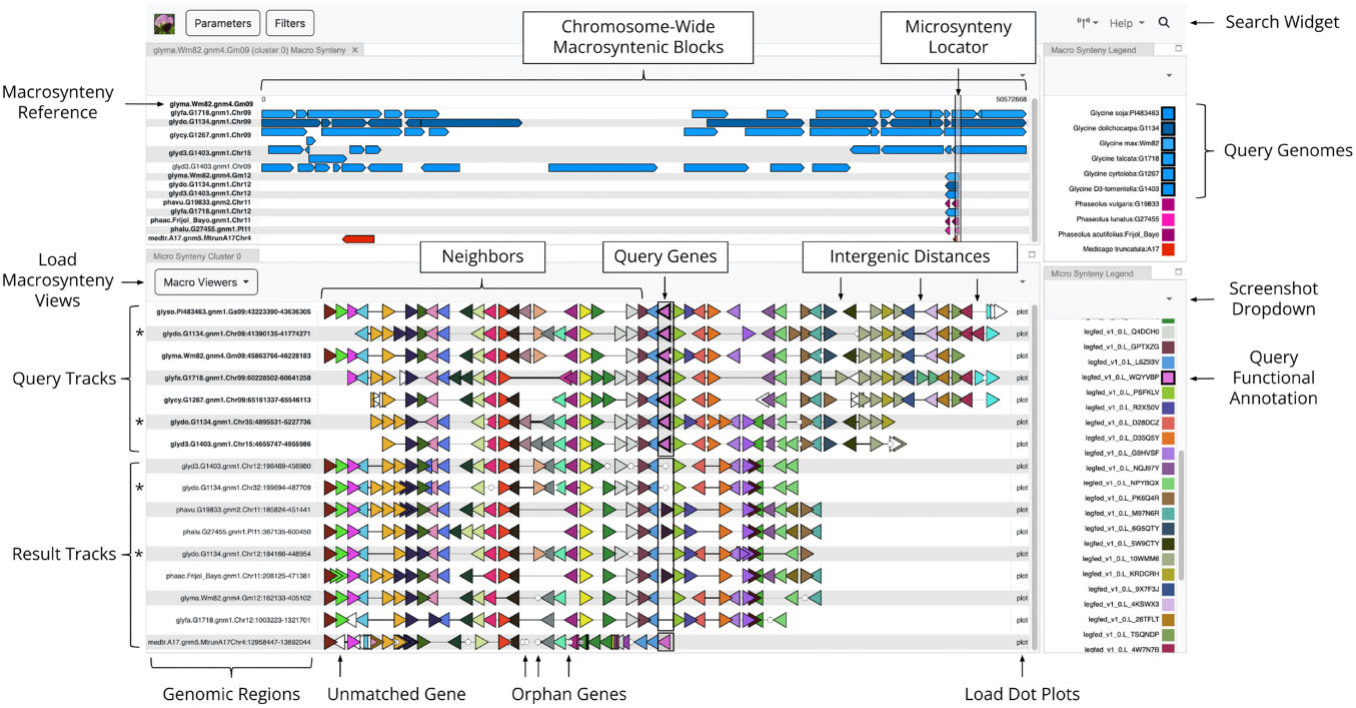
An example of GCV. *(bottom-left)* The microsynteny view depicts microsyntenic structures at the genic resolution. Each triangular glyph represents a gene, the orientation of which depicts the strand and the color depicts its functional annotation. White genes with a solid outline are the only gene present with a particular functional annotation. Orphan genes with a dashed outline do not have a functional annotation. The thickness of the lines between consecutive genes represents intergenic distance. The genes in the center with the bold outlines are the query genes. The genes that flank query genes are their neighbors. The label to the left of a track conveys the genomic region of the track. Query track labels are bold. *(bottom-right)* The functional annotations used to color the genes in the microsynteny and dot plot views. Annotations with bold outlines are those of the query genes. *(top-left)* The macrosynteny view depicts chromosome-wide macrosyntenic blocks relative to the chromosome of a microsynteny query track. Each directed block depicts a macrosynteny block, the orientation of which depicts the relative orientation of the chromosomes and the color depicts its non-query genome. Blocks are drawn relative to their position on the query chromosome, the extent of which is shown above the blocks. The label to the left of a track conveys the genome and chromosome of the track, with the query chromosome label at the top. Chromosomes that appear in the microsynteny view have bold labels. The semiopaque bar that vertically spans multiple tracks highlights the region of the query chromosome’s microsynteny track and the blocks it is composed of. *(top-right)* The genomes used to color the blocks in the macrosynteny and Circos views. Genomes with bold outlines are those of the microsynteny query tracks. *(not shown)* Pipelines, dot plots, and the Circos view. *(biology)* The biology of this GCV is discussed in the Examples section. The query annotation (pink and outlined in bold) is the ethylene responsive transcription factor (ERF) gene family. Aligned occurrences of the ERF gene family in the microsynteny view are highlighted with a black border and semiopaque fill. Occurrences of the ERF gene being substituted or deleted in the alignments are highlighted with a black border and transparent fill. The microsynteny tracks resulting from the *G. dolichocarpa* whole genome duplications are marked with an asterisk (*).

GCV’s primary function is computing and visualizing microsynteny. In the microsynteny view, segments of chromosomes are drawn as *tracks* composed of sequences of genes colored by their functional annotation. Given a microsynteny query track, GCV will search for tracks with similar functional annotation content and use local alignment algorithms to draw the result tracks in a manner that visually emphasizes preservation and variation of structure relative to the query track. Macrosynteny can be computed for the chromosomes in the microsynteny view and visualized as either synteny blocks relative to the query chromosome or as an all-pairs Circos style view (4). Both macrosynteny views color blocks by their genome. Local and global gene locus dot plots can also be computed for pairs of tracks in the microsynteny view, where *local* dot plots contain gene pairs for a query track and a result track, and *global* dot plots contain gene pairs between any gene on a query track’s chromosome and a search result track. Figure 1 shows an example of GCV and describes its anatomy.

GCV supports loading data from multiple databases, or *data federation*. By using a common set of functional annotations, GCV can compute synteny between genomes from otherwise unrelated databases on-demand in real-time. What databases GCV loads data from is configured by site administrators when they deploy GCV.

### Inputs

To build a query track, GCV must be provided a *query gene* on which the track will be centered and the number of *neighbor* genes that should flank the query gene on either side. Alternatively, an interval on a chromosome can be provided from which GCV will infer the query gene and number of neighbors. In both cases, what database to load the track from must be specified, and the name of the query gene or chromosome must exactly match a gene or chromosome in the database, respectively. These inputs are given to GCV via its URL and are intended to be provided by another application or GCV itself. For instance, a new query track can be derived from a specific gene or track in a GCV microsynteny view, or multiple query tracks can be derived from a functional annotation’s set of genes in a microsynteny view. Similarly, a new chromosome interval can be derived from a chromosome in either of the macrosynteny views. An annotated microsynteny query track is shown in Figure 1.

Alternatively, users can load tracks directly in GCV using the search widget. This widget is in the navigation bar present on every page in GCV. To use the widget, the user inputs one or more gene names or chromosome regions they would like to search for and optionally select which databases they would like to search; every database will be searched by default. The search results page will display all genes and chromosomes in the selected databases that approximately matched the user’s input. From here the user can select one or more genes or chromosome regions and use them as microsynteny query tracks by specifying the number of neighbors.

### Outputs

The primary output of GCV is its visualizations. The microsynteny view elucidates structural conservation and variation that may have functional significance, the macrosynteny view puts the microsyntenic structures into context by highlighting what macrosyntenic structures they occur in, and the dot plots provide a more intuitive representation of the physical distance between genes in the microsynteny view. A Scalable Vector Graphics (SVG) image of each view can be downloaded for inclusion in publications via a dropdown menu in the upper right corner of each view.

The secondary ‘output’ of GCV is its ability to interact with itself and other web applications, as described below.

### Interactivity

All GCV views are interactive. Mousing over an element in any view will highlight the element and all related elements in other open views. Additionally, if GCV’s broadcast channel feature is enabled, this will also highlight related elements in instances of GCV running in other web browser windows, allowing users to explore multiple related contexts simultaneously. Clicking on an element in a view will open a menu. Depending on the element, a new microsynteny view derived from the element may be loaded from this menu. GCV may also be configured to provide links in this menu to other web applications related to the clicked element.

### Configuration

GCV is highly configurable by site administrators. As previously mentioned, GCV can be configured to load data from multiple databases. Additionally, the navigation bar logo and instructions page screenshots and text can be customized for a particular site. GCV can also be configured to load data from optional services, such as a linkout service that will provide external links for a clicked element based on what type of element was clicked and its properties. This functionality can be used to integrate GCV with other web applications.

GCV can also be configured at run-time via algorithm parameters. These parameters control how GCV searches for and aligns microsynteny tracks and how macrosynteny tracks are computed. The parameters can be controlled by the user through the user interface or through GCV’s URL query string parameters. Moreover, GCV’s algorithm parameters and query track information is always kept up-to-date in the URL. This allows a specific state of GCV to be bookmarked or shared with collaborators.

### Version 2 Updates and New Features

Version 1 of GCV was reported in (3). Version 2 has brought significant updates to both the web application and the database software that supports it. Here we briefly highlight updates and new features that enhance usability, performance, and ease of deployment.

In the web application, chromosome regions can now be used as query tracks. Users can also now provide multiple query tracks for a single microsynteny view. In this case, GCV first clusters the query tracks and then searches for tracks similar to each cluster. The query tracks within a cluster are multiple aligned for visualization and the search result tracks are locally aligned to the multiple alignment’s consensus.

Macrosynteny is now computed on-demand, whereas in version 1 it was pre-computed. This new approach allows macrosynteny to be computed consistently across databases in a federated manner. Subsequently, a Circos style view that depicts all-pairs macrosynteny among the query chromosomes has been added. In the original macrosynteny view, macrosynteny blocks can now be ordered by syntenic distance. Additionally, a *pipeline* element has been added to every view to better convey the purpose of a view, how it functions, and what its current status is.

The main user interface in the web application has been updated to use a dynamic layout framework. Unlike version 1, which had a fixed number of views, this allows many arbitrary views to be loaded simultaneously and the user has complete control over their placement and size on the screen.

The web application can now optionally send messages based on user interactions over a JavaScript Broadcast Channel chosen by the user. This allows user interactions to propagate to other web applications and instances of GCV running in other web browser windows.

Lastly for the web application, a search widget was added to the navigation bar to drive a new search page. This allows users to perform a text search for genes and chromosome regions from which they can build microsynteny query tracks. In version 1, users had to know the exact name of the gene they wanted to build a query track from.

In the database software, the database was migrated from PostgreSQL with a Chado schema (5) to a custom Redis database, which greatly improved run-time performance. The new database can be loaded from a Chado database or directly from GFF and tab-delimited gene family assignment files. The database software itself was migrated from a monolith to the microservices architecture. Each individual service is containerized and published on the GitHub Package Registry. Version controlled Docker Compose files are available to allow GCV to be easily deployed using these containers.

## RELATED WORK

There are several web applications worth comparing with GCV. Traditional genome browsers, like JBrowse (6) and the UCSC Genome Browser (7), can complement GCV by providing a sequence level view of regions of interest identified within GCV. These views can be further enhanced by overlaying additional annotations not shown in GCV. Gene family trees, like those of Phytozome (8), can also complement GCV by enabling users to assess the function of genes identified within GCV.

There are also several web applications with functionality similar to GCV. Applications similar to GCV’s microsynteny view include MicroSyn (9), Phytozome’s gene family synteny view (8), Genomicus’ AlignView (10), Plaza’s Synteny and Multiplicon plots (11), PGDD’s colinear block view (12), MGcV’s comparative context map (13), and CoGe’s GEvo (14). Of these applications, only Phytozome, Genomicus, Plaza, and MGcV compute microsynteny using functional annotations, and only Genomicus aligns the genes among the microsynteny tracks. Genomicus microsynteny tracks, however, only contain gene families present in the anchor track, i.e. the other tracks may not depict the complete gene content in their chromosome segments. Additionally, Genomicus breaks proximal structural events into multiple tracks. Conversely, GCV microsynteny tracks contain every gene from their chromosome segments and proximal structural events are drawn in a single track wherever possible to make the proximity visually apparent.

There are few web applications with functionality similar to GCV’s microsynteny search. These include PGDD’s Synteny Search and CoGe’s SynFind (15). Like GCV, given a gene or region these applications will find similar microsyntenic regions. The other previously mentioned microsynteny applications simply build tracks statically, e.g. around all genes in a particular gene family. GCV can do this too, but will then also search for tracks similar to the built tracks. The search result tracks need not contain the functional annotation of the genes from which the query tracks were built.

Web applications similar to GCV’s macrosynteny view include Cinteny (16), CoGe’s SynMap (17), Gramene’s Synteny Maps (18), and MultiSyn (19). Of these, only Cinteny and Gramene compute synteny using annotations, only Cinteny does computations on-demand, and only MultiSyn can display synteny among several genomes simultaneously. Conversely, using functional annotations GCV can compute macrosynteny on-demand among several genomes.

Most critically, none of these web applications provide an integrated, multi-resolution interface like GCV, let alone where all the computations are done on-demand in real-time. Additionally, none of the applications support loading data from multiple databases. Not only can GCV load data from multiple databases, this functionality also enables users to run GCV locally with their own data while loading data from other public databases. Furthermore, GCV’s high level of interactivity surpasses that of the other tools and enables users to rapidly explore synteny within GCV and through interaction and integration with other web applications.

## METHODS

GCV is a menagerie of algorithms. Here we describe those behind GCV’s primary functionality.

### Query tracks

Query tracks are clustered via hierarchical agglomerative clustering using the Levenshtein distance. Query tracks within the same cluster are multiple aligned for visualization by profile Hidden Markov Model (20).

### Microsynteny search

We formulate microsynteny search as an instance of the Fixed-Radius Near Neighbors problem (21) and implemented it using an Gaps-and-Islands algorithm.

### Microsynteny track alignment

Microsynteny search result tracks are aligned to the query track (or the consensus track if multiple queries are present) using the Smith-Waterman (22) and Repeat (23) local alignment algorithms. Inverted segments are included in alignments using a weighted interval scheduling algorithm (24). Aligned tracks are drawn without overlaps using the greedy interval scheduling algorithm (25).

### Macrosynteny

Macrosynteny is computed on-demand using a simplified MCScanX style algorithm (26). We have previously verified that the blocks computed using our algorithm are approximately the same as those computed with MCScanX when using functional annotations to define gene homologues (27). The metric used to order macrosynteny blocks is a 2-mer multiset Jaccard distance that supports inversions. Like aligned microsynteny tracks, a chromosome’s macrosynteny blocks are drawn without overlaps using the greedy interval scheduling algorithm.

### Data federation

Federation is achieved by databases deploying the GCV microservices with their own genomes annotated with the same functional annotations used by other databases. GCV then performs federated computations by sending ordered lists of functional annotations to the services. These ordered lists of annotations can represent either microsynteny tracks or entire chromosomes.

## USE CASES AND EXAMPLES

GCV is developed by the Legume Information System (LIS) and has been part of the LIS website since 2013 (28). GCV is actively developed and continuously tested on the entire collection of LIS genomes. In this section we discuss the use cases enabled by GCV’s features and provide examples from the LIS GCV. Non-legume examples are available in the Wiki on GCV’s GitHub repository. See Table 1 for links to these examples and the LIS GCV.

**Table 1.** GCV URLs

GCV source code	https://github.com/legumeinfo/gcv
Apache 2.0 software license	https://www.apache.org/licenses/LICENSE-2.0
GCV examples	https://github.com/legumeinfo/gcv/wiki/Examples
GCV at LIS	https://gcv.legumeinfo.org
GCV at Araport	https://gcv-arabidopsis.ncgr.org
GCV at SoyBase	https://gcv.soybase.org
GCV for Medicago genus-wide pangenome	https://medicago.legumeinfo.org/tools/gcv
GCV for Phaseolus genus-wide pangenome	https://phaseolus.legumeinfo.org/tools/gcv
GCV for Vigna genus-wide pangenome	https://vigna.legumeinfo.org/tools/gcv

### Use cases

The primary use case of GCV is its microsynteny search. This has not changed in version 2 of GCV, but rather has been greatly enhanced by the support for multiple query tracks. Specifically, a user can now perform a microsynteny search for multiple segments that are already known to be syntenic, yielding a set of result tracks that better corresponds to the variation within the query tracks, such as when working with pangenomes. By clustering the query tracks prior to performing a microsynteny search, users may also search using a more disparate set of query genes, such as genes that share a functional annotation but the contexts in which they occur are unknown. This provides the benefits of multi-query track search to each cluster and, using the dynamic layout framework introduced in version 2, allows users to compare the clusters and their search results within a single user interface. This is especially useful for identifying large structural events and investigating neofunctionalization.

GCV uses macrosynteny to complement the microsynteny search by showing the macrosyntenic structures in which the microsyntenic structures occur. This is especially useful for revealing the extent of structural events and identifying other microsyntenic regions of interest. Similar to microsynteny search, this has not changed in version 2 of GCV, but rather has been enhanced by computing macrosynteny on demand, instead of using precomputed data, as in version 1. By computing macrosynteny on-demand, all genomes accessed through the microsynteny search will also have corresponding macrosynteny structures. Also, like the microsynteny search, macrosynteny can now be computed in a federated manner by computing blocks for chromosomes from different databases. A new use case this has enabled is the all-pairs comparison of macrosynteny blocks using a Circos style view. In this view users can compare the synteny blocks of all chromosomes of the microsynteny query tracks simultaneously, allowing them to rapidly assess the conservation and variation of macrosyntenic structure among the chromosomes.

### Examples

Plant genomes have a remarkable ability to tolerate gene content modifications occurring as both whole genome duplication events (polyploidy) and smaller scale structural variations that can impact both gene content (PAV/CNV) and context (inversion/translocation). Some classes of gene families, such as those involved in plant responses to biotic and abiotic stress, are particularly subject to this type of content variation, while the retention of duplicated copies of genes following large scale duplication events can suggest a variety of mechanisms for imposing selective constraints on the apparent redundancy, such as sub- or neo-functionalization (29,30). GCV enables rapid exploration of such gene-level correspondences across phylogenetic distances whose scale can be varied to suit the nature of the question at hand through use of gene families described at different levels of taxonomic resolution.

An example is given in Figure 1, where a set of genes from soybean (31) and several wild perennial *Glycine* relative species (32) were used as queries. All of these species share a recent (10–13 million years ago) whole genome duplication (WGD) event not present in other legume species, and *G. dolichocarpa* (glydo) has undergone an additional recent WGD (33). The query genes all belong to the same ethylene responsive transcription factor (ERF) gene family (legfed_v1_0.L_WQYVBP), and a neighborhood of 22 genes on either side of the query genes was retrieved per user-specified parameters. The profile HMM used to construct a multiple alignment of these tracks also served as the basis for a search for other segments with similar functional annotation content (result tracks need not have a copy of the query gene family). Although many species in the database have regions matching the criteria, here the result tracks are filtered to include only the same species as the query tracks plus several outgroup species: *M. truncatula* (medtr), *P. vulgaris* (phavu), *P. lunatus* (phalu), and *P. acutifolius* (phaac). These species do not share the WGD and help to infer the ancestral state of the genomic regions shown. In the case of the gene family of interest, we see that the more distantly related *M. truncatula* also has a copy of the gene at this location, while the *Phaseolus* species have all substituted the ERF for a gene in a different relative orientation and assigned to a different family. The result tracks also include homoeologous segments from the various *Glycine* species, in which the ERF gene is strikingly absent, despite substantial matched content surrounding the query genes. This can be contrasted by the state of the gene two places to the right of the query genes, where retention and loss of the copy at that position occur scattered between the orthologous query tracks and the homoeologous result tracks.

Another significant observation that can be made from the view presented is the fact that while the result tracks match the neighbors on the left of the query genes, they fail to cover the entire neighborhood on the right hand side. The reason for this can be seen in the macrosynteny view, where it is apparent that the orthologous *Glycine* chromosomes designated as 09 have macrosynteny across the entire extent of the chromosomes (with the exception of the areas around the center, which tend to be repeat-rich and gene-poor), while the homoeologous chromosomes designated as 12 have only a small block of synteny whose breakpoint is being straddled on one side by the microsynteny region. The orthologous regions in the outgroup species are similarly limited, suggesting that the chromosome 12 configuration is likely to be closer to the ancestral state. We also see that in the *Glycine* species *G. tomentella sensu lato* D3 (glyd3) a chromosomal rearrangement relative to the other *Glycine* species has occurred such that the orthologous microsyntenic region is located on a chromosome named 15, with the chromosome having a larger extent of macrosynteny to the soybean Gm09 having been designated Chr09 in this species.

It is also worth noting that in this example all the query genes were taken from the segments which aligned well across the entire region. If copies of a family with members dispersed between the two groups of homoeologous segments had been selected (as with the members of the gene family two places to the right of the query), then depending on user-specified parameters for clustering the segments from the different groups might be split into two or more separate sets, each of which would define its own profile HMM and would potentially find different result sets, depending on parameters used to define the required similarity. Each cluster and corresponding results would be presented in a separate pane of the view and can have its own macrosynteny or dotplot views associated, while the family legend can still be used to highlight occurrences of gene families that are common across the clusters. The dynamic highlighting of gene family instances is also useful for clarifying the copy number of families within individual tracks and cases of local translocation in which a member is present in a microsyntenic view but not aligned with other instances of the family.

This example, including its URL and the list of query genes, is available on the Examples page in the Wiki on GCV’s GitHub repository. See Table 1 for a link to the Wiki.

## DATA AVAILABILITY

GCV is free and open-source software available on GitHub under the Apache 2.0 software license. A version containing representative genomes for crop and model legumes and wild relative species is available via the Legume Information System (LIS). LIS also houses a growing number of instances centered on pangenome collections for genera whose members have multiple genomes sequenced per species. A version containing various *Arabidopsis* and other Brassicaceae genomes is available via Araport (34). And a version containing multiple *Glycine max* and *soja* genomes is available via SoyBase (35). See Table 1 for links.
